# Dissection of exopolysaccharide biosynthesis in *Kozakia baliensis*

**DOI:** 10.1186/s12934-016-0572-x

**Published:** 2016-10-04

**Authors:** Julia U. Brandt, Frank Jakob, Jürgen Behr, Andreas J. Geissler, Rudi F. Vogel

**Affiliations:** Technische Universität München, Lehrstuhl für Technische Mikrobiologie, Gregor-Mendel-Straße 4, 85354 Freising, Germany

**Keywords:** Acetic acid bacteria, *Kozakia baliensis*, Exopolysaccharides, Genome analysis, Gum-like cluster, Heteropolysaccharides

## Abstract

**Background:**

Acetic acid bacteria (AAB) are well known producers of commercially used exopolysaccharides, such as cellulose and levan. *Kozakia (K.) baliensis* is a relatively new member of AAB, which produces ultra-high molecular weight levan from sucrose. Throughout cultivation of two *K. baliensis* strains (DSM 14400, NBRC 16680) on sucrose-deficient media, we found that both strains still produce high amounts of mucous, water-soluble substances from mannitol and glycerol as (main) carbon sources. This indicated that both *Kozakia* strains additionally produce new classes of so far not characterized EPS.

**Results:**

By whole genome sequencing of both strains, circularized genomes could be established and typical EPS forming clusters were identified. As expected, complete ORFs coding for levansucrases could be detected in both *Kozakia* strains. In *K. baliensis* DSM 14400 plasmid encoded cellulose synthase genes and fragments of truncated levansucrase operons could be assigned in contrast to *K. baliensis* NBRC 16680. Additionally, both *K. baliensis* strains harbor identical *gum*-like clusters, which are related to the well characterized *gum* cluster coding for xanthan synthesis in *Xanthomanas campestris* and show highest similarity with *gum*-like heteropolysaccharide (HePS) clusters from other acetic acid bacteria such as *Gluconacetobacter diazotrophicus* and *Komagataeibacter xylinus*. A mutant strain of *K. baliensis* NBRC 16680 lacking EPS production on sucrose-deficient media exhibited a transposon insertion in front of the *gumD* gene of its *gum*-like cluster in contrast to the wildtype strain, which indicated the essential role of *gumD* and of the associated *gum* genes for production of these new EPS. The EPS secreted by *K. baliensis* are composed of glucose, galactose and mannose, respectively, which is in agreement with the predicted sugar monomer composition derived from in silico genome analysis of the respective *gum*-like clusters.

**Conclusions:**

By comparative sugar monomer and genome analysis, the polymeric substances secreted by *K. baliensis* can be considered as unique HePS. Via genome sequencing of *K. baliensis* DSM 14400 + NBRC 16680 we got first insights into the biosynthesis of these novel HePS, which is related to xanthan and acetan biosynthesis. Consequently, the present study provides the basis for establishment of *K. baliensis* strains as novel microbial cell factories for biotechnologically relevant, unique polysaccharides.

**Electronic supplementary material:**

The online version of this article (doi:10.1186/s12934-016-0572-x) contains supplementary material, which is available to authorized users.

## Background

The production of exopolysaccharides (EPSs) is a common attribute of many bacteria. On the basis of their monomer composition, EPSs are divided into two groups, homo- (HoPS) and heteropolysaccharides (HePS). These EPSs can be used as an extracellular matrix, in form of slime for biofilm formation and protection, or in form of a pellicle, which leads to floating cultures of the surface of the media to increase aeration. Especially HePS have unique properties, since their complex, mostly branched structures are responsible for drastic viscosity increases of aqueous solutions already in low concentrations. This characteristic of HePS is exploited in the food and cosmetic industry (e.g. in sauces, dressings, tooth paste, lotions etc.) always on an empirical basis. Therefore, EPS forming bacteria receive high attention for biotechnological applications, particularly *Xanthomonas (X.) campestris*, which produces the commercially important and widely used polysaccharide xanthan [1]. Industrial applications of xanthan are widely diversified and include areas like, foods, cosmetics as well as oil recovery [2, 3].

As an alternative to the addition of EPS as ingredients of food, EPS can also be produced in situ upon food fermentation by deliberately added appropriate starter strains [4]. In the respective products, the safety of the used starter culture and its metabolic products are of great importance and limit in situ EPS production to non-pathogenic, naturally and traditionally food-associated bacteria (e.g. in yoghurt, sourdough, kefir or kombucha). One group of non-pathogenic, food-grade bacteria comprises acetic acid bacteria (AAB), which have important roles in food and beverage production, for example vinegar, kombucha or kefir [5].

AAB are well known for their ability to produce large amounts of EPSs, either homopolysaccharides, like dextrans, levans and cellulose, or different kinds of heteropolysaccharides [6], such as in the case of *Acetobacter (A.) tropicalis* [7], *A. aceti* [8], *Gluconacetobacter (Ga.) diazotrophicus* [9] and *Komagataeibacter xylinus* [10].

Heteropolysaccharides are formed in the cytoplasm via the sequential addition of nucleoside diphosphate sugars to growing repeating units. The assembly of the repeating unit is subsequently initialized via a so-called priming glycosyltransferase (GT), which loads the first sugar nucleotide to a C55 isoprenylphosphate lipid carrier anchored in the inner membrane [11]. This first sugar connected to the C55 carrier serves as acceptor of the next sugar nucleotide and so on, till the repeating unit is formed. The biosynthesis of these repeating units requires different kinds of GTs that are genetically encoded in organized clusters, which can reach up to 20 genes [12]. One distinct organization of such genes is known as the *gum*-cluster from *X. campestris*, involved in xanthan synthesis. This cluster includes all genes coding for GTs and enzymes necessary for the polymerization and secretion of xanthan [13, 14]. In Gram negative bacteria the secretion and polymerization of HePS producing bacteria is mostly performed via a Wzx/Wzy-dependent pathway, whereas Wzx acts as a flippase, catalyzing the transport across the membrane, and Wzy is involved in the assembly of the HePS [15]. In *X. campestris* these two enzymes are coded by the *gumE* (Wzy) and the *gumJ* (Wzx) gene [16].

Furthermore, a few AAB strains produce different types of EPSs simultaneously. This simultaneous production of different kinds of EPS could lead to significantly higher (mostly desired) rheological effects, via the combination of different qualities of high and low molecular weight EPSs. Only a few multiple EPS producing bacteria among *Acetobacteracea*e have been identified so far [10, 17, 18], whereas these new candidates could offer great commercial potential (e.g. as EPS producing starter cultures for food fermentations) due to their specialization on EPS production.

*Kozakia (K.) baliensis* is a relatively new member of the family of *Acetobacteraceae* and is already well known to produce high molecular weight levans from sucrose, which significantly improve the quality of breads [19] [20]. Throughout cultivation of two *K. baliensis* strains (DSM 14400, NBRC 16680) on sucrose deficient media, we found that both strains still produce high amounts of mucous, water-soluble substances, which could exhibit promising properties for diverse (food) biotechnological applications.

Therefore, we wanted to detect the basic sugar composition of these polymeric substances via sugar monomer analysis and identify responsible biosynthesis clusters and putative transferases catalyzing the incorporation of specific sugars via whole genome sequencing of both *Kozakia* strains. In this way, first insights should be got into (i) the basic composition of these EPS and (ii) their respective biosynthesis routes.

## Methods

### Strains, media and growth conditions

*Kozakia* strains (DSM 14400^T^, NBRC 16680) were screened for their ability to produce mucous substances on modified sodium-gluconate medium (NaG) agar (without sucrose) over a time span of 72 h at 30 °C. Both *K. baliensis* strains were generally cultivated aerobically at 30 °C in liquid NaG media (20 g/L sodium gluconate, 3 g/L yeast extract, 2 g/L peptone, 3 g/L glycerol, 10 g/L mannitol, pH adjusted to 6.0).

### General molecular techniques

For genome sequencing, genomic DNA was isolated following the instructions of the Qiagen Genomic DNA Kit (Qiagen, Hilden, Germany). The genomic DNA of *K. baliensis* strains DSM 14400 and NBRC 16680 were submitted to GATC Biotech (Germany) for PacBio single-molecule real-time (SMRT) sequencing, respectively. A single library was prepared for both strains, which were run on one SMRT cell, respectively. All generated sequences were assembled with a hierarchical genome-assembly process version 3 (HGAP3), including an assembly with the Celera Assembler and assembly polishing with Quiver [21]. Initial ORF predictions and annotations were accomplished automatically using the program RAST, a SEED-based, prokaryotic genome annotation service [22]. Annotations were corrected via the NCBI Prokaryotic Genome Annotation Pipeline. EPS cluster annotations were performed via homology searches against GeneBank/EMBL using the function BLASTP. Whole genomic sequence data of both *K. baliensis* strains (DSM 14400, NBRC 16680) have been deposited in GeneBank.

For genetic characterization of a mutant strain of *K. baliensis* NBRC 16680, genomic DNA of the mutant strain was isolated following the instructions of the E.Z.N.A. Bacterial DNA Kit (Omega Biotek, Norcross, USA). DNA sequences were amplified using Taq-DNA-Polymerase (Qbiogene, USA) or KAPA HiFi PCR Polymerase (Peqlab, Erlangen, Germany). Primers used for PCR reactions are listed in Additional file 1: Table S1. Restriction endonuclease digestions were performed as recommended by the suppliers (Fermentas, St. Leon-Roth, Germany). Following restriction enzymes were used: HpaI, ApaI, DraI, SalI, XhaI; SacI, AgeI, EcoRI, EcoRV, HindIII, NheI, and SmaI. PCR products were sequenced via sanger sequencing by GATC Biotech (Konstanz, Germany). Preparative DNA isolations from agarose gels were performed with the peqGOLD Gel extraction Kit (Peqlab Erlangen, Germany), PCR products were purified using the E.Z.N.A. Cycle-Pure Kit (Omega Bio-tek, Norcross, USA).

### EPS production and isolation

To isolate EPS samples from liquid media, the respective strains were grown in 10 mL of NaG media and incubated for 32 h on a rotary shaker (200 rpm) at 30 °C. After cell removal, EPS in the supernatant was precipitated with cold ethanol (2:1, v/v) and kept overnight at 4 °C. This step was repeated three times, followed by a dialysis step (MWCO 14 kDa) of the recovered (centrifugation) and in ddH_2_O re-dissolved EPS. Finally, the purified HePSs were lyophilized and quantified by weighing.

### Determination of sugar monomers in isolated EPS samples

The monosaccharide composition of isolated EPS was investigated via high performance liquid chromatography (HPLC). The purified polysaccharide samples were hydrolyzed with 15 % of perchloric acid (70 %) over 7 h at 100 °C, followed by a centrifugation step (4 °C, 10 min, 13,000*g*) for removal of possible impurities such as proteins. The supernatant was analyzed using a Rezex RPM column (Phenomenex, Germany) coupled to a refractive index (RI) detector (Gynkotek, Germany) according to the method of [23]. Sugar monomers were identified according to their retention time using suitable monosaccharide standards (d-glucose, d-galactose, d-mannose, d-rhamnose). The mobile phase was water, with a flow rate of 0.6 mL/min.

### Comparison of pellicle and EPS production between *K. baliensis* NBRC 16680 wildtype and mutant strain

To investigate surface pellicle production of an EPS deficient mutant strain of *K. baliensis* NBRC 16680, 5 mL of static cultures were inoculated with 50 µl of overnight cultures (wildtype and mutant, respectively) and cultivated statically at 30 °C for 3 days. Pellicle production was observed and documented macroscopically. Furthermore, EPS production in shaking cultures was investigated via cultivation of the mutant strain of *K. baliensis* NBRC 16680 over 32 h in NaG media. Isolation and quantification of EPS was performed according to the described EPS isolation (see above).

## Results

### Analysis of EPS composition and production

**Fig. 1:**
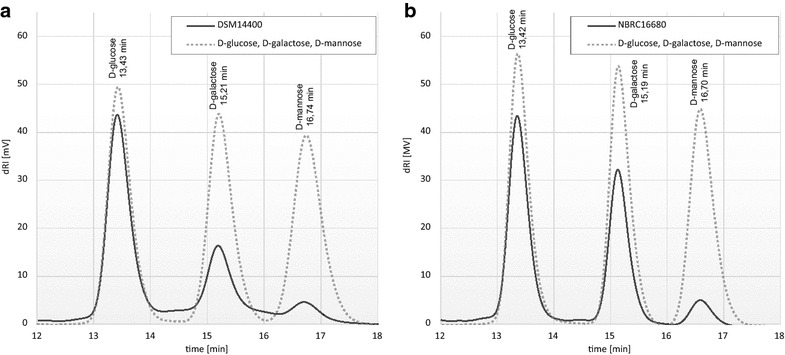
Monomer composition of the HePS isolated from *K. baliensis* strains DSM 14400 and NBRC 16680. HPLC profiles of the perchloric acid hydrolysates of HePS from *K. baliensis* DSM 14400 (**a**) and *K. baliensis* NBRC 16680 (**b**) in comparison with a 25 mM standard mix (*dotted line*) composed of d-glucose (13,43 min), d-galactose (15,21 min) and d-mannose (16,74 min). The retention times of the detected sugar monomers in the respective acid hydrolysates were consistent with the retention times of the standards

For EPS extraction, *K. baliensis* strains (DSM 14400, NBRC 16680) were grown for 32 h in 10 mL of NaG liquid medium without sucrose. After freeze-drying of the isolated EPS, the amount was measured by weight, which resulted in about 1.87 ± 0.04 g/L (DSM 14400) and 1.71 ± 0.05 g/L (NBRC 16680) of EPS for each strain. Both isolated EPS showed good solubility in water after freeze-drying. Concentrations of ~5 g/L (0.5 % aqueous solution) resulted in drastic viscosity increases. In the HPLC pattern of the perchloric acid hydrolysate of both *K. baliensis* EPS, three peaks could be observed (Fig. 1). The retention times of the detected monomers were consistent with the retention times of the standards d-glucose (Glc), d-galactose (Gal) and d-mannose (Man).

### Global genome properties of *K. baliensis* DSM 14400 and NBRC 16680

**Fig. 2:**
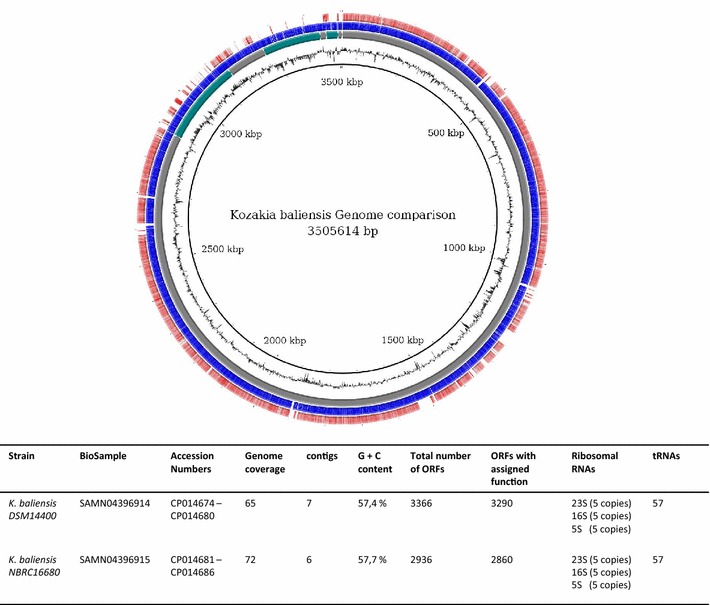
Genome comparison and general features of *K. baliensis* strains DSM 14400 and NBRC 16680. Starting from inside:* circle 1* shows the general genomic position in kilobases;* circle 2* depicts the varying G+ C-content of *K. baliensis* DSM 14400 at different genetic loci;* circle 3* is composed of the seven contigs of *K. baliensis* DSM 14400 [main chromosome and additional (partial) plasmids];* circle 4* reflects the coding density of *K. baliensis* DSM 14400;* circle 5* shows the blast identities (*red*) of *K. baliensis* NBRC 16680 in comparison to *K. baliensis* DSM 14400 (note the low identity in plasmid regions)

Both *K. baliensis* genomes were sequenced via PacBio single-molecule real-time (SMRT) sequencing. De novo assembly was carried out by using the hierarchical genome assembly process (HGAP) method [21]. For *K. baliensis* DSM 14400 seven contigs were generated upon sequencing, which were further assembled to one finished genome. The genome consists of one circularized chromosome (2,888,029 bp) and four circularized plasmids designated as pKB14400_1 (253,508 bp), pKB14400_2 (102.455 bp), pKB14400_3 (176,911 bp) and pKB14400_4 (18,625 bp), as well as two partial plasmids [pKB14400_5 (35.463 bp), pKB14400_6 (30.533 bp)]. Genome sequencing of *K. baliensis* NBRC 16680 resulted in six contigs, which were assembled to one chromosome (2,807,246 bp) and three plasmids: pKB16680_1 (135,727 bp), pKB16680_2 (80,099 bp) and pKB16680_3 (36,022 bp), as well as two partial plasmids [pKB16680_4 (14.635 bp), pKB16680_5 (11.848 bp)]. The average GC content of the DSM 14400 strain is 57.4 %, and 57.7 % for the NBRC 16680 strain. Both *Kozakia* chromosomes show high conformity, the average nucleotide identities are 98 % within a query coverage of 94 %. The plasmid identity is low up to unique plasmids in *K. baliensis* DSM 14400 (Fig. 2).

### Clusters coding for putative HePS biosynthesis

**Fig. 3:**
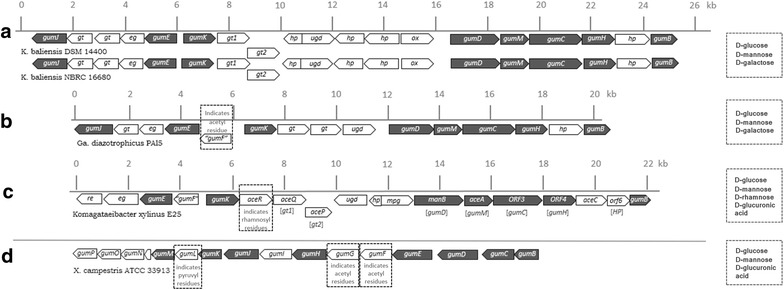
Genetic organization of HePS biosynthesis encoding *gum*-like clusters. The *gum*-like clusters of *K. baliensis* strains DSM 14400 + NBRC 16680 are depicted in (**a**). The cluster has an overall size of ~25 kb and involves 19 genes, including glycosyltransferases (*gt*), hypothetical proteins (*hp*), and eight *gum* like genes (*gumB,* -*C,* -*D,* -*E,* -*H,* -*J,* -*K, and* –*M*), which are marked in* grey*. Furthermore, the cluster contains a putative endoglucanase (*e.g.*), oxidoreductase (*ox*) and a UDP-glucose dehydrogenase (*ugd*). **b**, **c** show the related *gum* like clusters of the AAB strains *Ga. diazotrophicus* PAI5 and *Komagataeibacter xylinus* E25. The *Ga. diazotrophicus* cluster exhibits, in comparison to both *K. baliensis* clusters, an additional *gumF* gene, that could putatively incorporate acetyl-residues at specific positions into the related HePS. The so called acetan cluster of *Ko. xylinus* harbors—besides an additional *gumF* gene—a rhamnosyl transferase, coded by *aceR*, as well as a mannose-phosphate-guanyltransferase (*mpg*). The nomenclature for the acetan cluster in (**c**) is based on Griffin AM, Morris VJ and Gasson MJ [44], while brackets under the particular genes mark the homologous *gum* genes. In (**d**) the *gum* cluster of *X. campestris,* which consists of *gumB,* -*C,* -*D,* -*E,* -*F,* -*G,* -*H,* -*I,* -*J,* -*K,* -*L,* -*M,* -*N and* –*P*, is depicted. The* dotted squares* in the particular *gum*-clusters of B, C and D mark genes, which alter between the specific clusters (relative to both *K. baliensis* gum-like clusters). The corresponding monomer compositions of the respective HePSs are shown at the* right*. The putative functions of the corresponding annotated genes are as follows (derived from Pühler et al. [26] and Griffin et al. [45]): *aceA* UDP-glucosyltransferase, *aceC* GDP-mannosyltransferase, *aceP* glucosyltransferase, *aceQ* glucosyltransferase, *aceR* rhamnosyltransferase, *gumJ* export protein, *gumE* polymerization or export protein, *gumK* catalyzes the addition of glucuronic acid, *gumD* catalyzes the addition of glucose-1-phosphate, *gumM* catalyzes the addition of glucose in β-1,4-position, *gumC* polymerization and export protein, *gumG* acetyl transferase, *gumL* pyruvyl transferase, *gumH* catalyzes the addition of internal mannose, *gumI* β-mannosyltransferase, *gumB* polymerization and export protein, RE dTDP-4-dehydrorhamnose 3,5-epimerase, *manB* Mannose-1-phosphate guanylyltransferase, *tp* transporter

In both *Kozakia* genomes typical clusters for putative HePS production were identified, including a HePS gene cluster of 25 kb (“*gum*-like cluster”, Fig. 3) and a “*pol*-cluster” (Fig. 5b), which comprises the genes *polABCDE* and was previously shown to be involved in pellicle formation in *Acetobacter tropicalis* via the biosynthesis of capsular HePS [24]. The genetic organizations of the *gum*-like clusters of both *K. baliensis* strains are depicted in Fig. 3a and exemplarily compared to *gum*-like clusters of *Komagataeibacter (Ko.) xylinus* E25 (Fig. 3b) (formerly *Ga. xylinus*), *Ga. diazotrophicus* PA15 (Fig. 3c) and *X. campestris* (Fig. 3d). While the *gum*-like clusters of *K. baliensis* share identical genetic organizations among each other, they are next related to *gum*-like clusters from the AAB strains *Ko. xylinus* and *Ga. diazotrophicus*. On the contrary, the genetic organization of the gum-cluster of *X. campestris* differs remarkably from those of the depicted AAB strains (Fig. 3), while the gum proteins of *X. campestris* exhibit principal homology to those of the depicted AAB strains (Table 1). The *gum*-like clusters of both *Kozakia* strains involve in total 19 ORFs, which are mainly designated as glycosyltransferases with unknown function or hypothetical proteins (11 genes), and eight proteins, which show homology to the well-characterized *gum*-proteins catalyzing xanthan biosynthesis in *X. campestris.* These homologous gum-like genes are *gumB,* -*C,* -*D,* -*E,* -*H,* -*J,* -*K,* and –*M* (Fig. 3, Table 2). The *gumD* gene is described to catalyze the first step of the HePS synthesis, by transferring the first sugar-1-phosphate to an undecaprenyl-phosphate-lipid carrier in the membrane [25]. *gumH, gumK* and *gumM* encode glycosyltransferases, which are involved in the sequential transfer of mannosyl-1-phosphate, glucosyl-1-phosphate and glucuronyl acid 1-phosphate residues (GlcA), from activated sugar nucleotides, including UDP-glucose, UDP-glucuronic acid and GDP-mannose, respectively [26, 27]. *GumE* and *gumJ* are assigned to have a function during the polymerization and translocation of the repeating units [26, 28]. *GumB* and *gumC* share sequence similarities to the *Escherichia* (*E.*) *coli* proteins Wza and Wzc and therefore could be involved in the export of the HePS [29, 30]. Not all genes of the *gum*-operon from *X. campestris* are present in *K. baliensis* (*gumF*, -*G*, -*I* and –*L*, Fig. 3). These genes are *inter alia* associated with the incorporation of acetyl- and pyruvyl-residues into the xanthan backbone [13].

**Table 1:** Homology comparison between Gum proteins of *K. baliensis* DSM 14400 and Gum proteins of HePS biosynthesis related bacteria

Kozakia baliensis DSM 14400	Kozakia baliensis NBRC 16680	Gluconacetobacter diazotrophicus PAI 5	Komagataeibacter xylinus E25	Xanthomonas campestris str. ATCC 33913
Protein	Protein sequence identities & query cover	Protein sequence identities & query cover	Protein sequence identities & query cover	Protein sequence identities & query cover
GumJ A0U89_05160;502aa	498/502 (99 %); 100 % A0U90_06730 “GumJ”	322/510 (63 %); 98 % GDI2535 “GumJ”		102/456 (22 %); 96 % XCC2446 GumJ
GumE A0U89_05140;416aa	410/416 (99 %); 100 % A0U90_06750 “GumE”	226/393 (58 %); 94 % GDI2538 “GumE”	203/411 (49 %); 98 % H845_814 “GumE”	96/332 (29 %); 74 % XCC2451 GumE
GumK A0U89_05135;373aa	370/373 (99 %); 100 % A0U90_06755 “GumK”	244/369 (66 %); 98 % GDI2542 “GumK”	244/369 (66 %); 98 % H845_816 “GumK”	162/369 (44 %); 91 % XCC2445 GumK
GumD A0U89_05095;420aa	414/420 (99 %); 100 % A0U90_06795 “GumD”	239/420 (57 %); 100 % GDI2547 “GumD”	234/474 (49 %); 94 % H845_822 “AceA” (Griffin,1994)	159/486 (33 %); 96 % XCC2452 GumD
GumM A0U89_05090;271aa	268/271 (99 %); 100 % A0U90_06800 “GumM”	175/253 (69 %); 92 % GDI2548 “GumM”	137/248 (55 %); 91 % H845_823 “ORF3” (Griffin,1994)	66/230 (29 %); 85 % XCC2443 GumM
GumC A0U89_05085;722aa	716/722 (99 %); 100 % A0U90_06805 “GumC”	351/708 (50 %); 97 % GDI_2549 “GumC”	319/709 (45 %); 95 % H845_824 “ORF4” (Griffin,1994)	111/446 (25 %); 95 % XCC2453 GumC
GumH A0U89_05080;341aa	332/341 (99 %); 100 % A0U90_06810 “GumH”	223/370 (60 %); 98 % GDI2550 “GumH”	190/341 (56 %); 99 % H845_825 “AceC” (Griffin,1994)	162/372 (44 %); 97 % XCC2448 GumH
GumB A0U89_05070;189aa	189/189 (99 %); 100 % A0U90_06820 “GumB”	107/179 (60 %); 94 % GDI2552 “GumB”	102/183 (56 %); 95 % H845_828 “GumB”	50/145 (34 %); 66 % XCC2454 GumB

**Table 2:** Overview of *gum*-genes and their corresponding predicted protein functions involved in xanthan biosynthesis in *X. campestris* [26]

The presence of homologous *gum*-genes in *K. baliensis*, *Ga. diazotrophicus* and *Ko. xylinus* is indicated by white arrows

Via comparison of the genetic organizations and protein homologies of the gum-like clusters (Fig. 3, Table 1), the gum-like clusters of *K. baliensis* are most related to that of *Ga. diazotrophicus*, which produces a HePS composed of Glc, Gal, Man in approximate ratios of 6:3:1 [31]. This is in agreement with the detected sugar monomers in the isolated HePS of *K. baliensis* DSM 14400 and NBRC 16680 (Fig. 1). Though, there are different genes in *K. baliensis*/*Ga. diazotrophicus* gum clusters (e. g “*gumF*” putatively incorporating acetyl residues, Fig. 3b). Furthermore, a homology comparison between the previously described acetan biosynthesis cluster from *Ko. xylinus* E25 and the gum-like clusters of *K. baliensis* revealed several “*ace*-*genes*” to be homologous to the gum-like genes of *Kozakia* (Fig. 3c, Table 1).

**Fig. 4:**
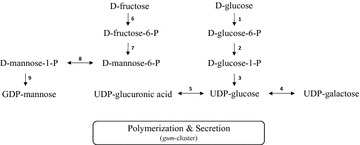
Schematic representation of the proposed nucleotide sugar biosynthesis related to EPS production in *K. baliensis*. Starting with the phosphorylation of fructose to fructose-6-phosphate (6) or glucose to glucose-6-phosphate (1), these intermediates can be converted into mannose-6-phosphate (7) or glucose-1-phosphate (2), respectively. Mannose-6-phosphate can be further converted into mannose-1-phosphate (8) and finally into GDP-mannose. UGP (3) catalyzes the synthesis of UDP-glucose from glucose-1-phosphate. UDP-glucose can be further isomerized to UDP-galactose (4) or UDP-glucuronic acid (5). The proposed pathway for the biosynthesis of activated nucleotide sugar precursors is based on publications from Kornmann et al. [18] and Pühler et al. [26]. The corresponding genomic locations of these respective genes are listed in Additional file 2: Table S2: *gk*-gene coding for a Glucokinase (EC 2.7.1.2); 2: *pgm*-gene coding for a Phosphoglucomutase (EC 5.4.2.2); 3: *ugp*-gene coding for an UDP-glucose-1-phosphate uridylyltransferase (EC 2.7.7.9); 4: *galE*-gene coding for an UDP-glucose-4-epimerase (EC 5.1.3.2); 5: *ugd*-gene coding for an UDP-glucose dehydrogenase (EC 1.1.1.22); 6: *fk*-gene coding for a Fructokinase (EC 2.7.1.4); 7: *mpi*-gene coding for a Mannose-6-phosphate isomerase (EC 5.3.1.8); 8: *pmm*-gene coding for a Phosphomannomutase (EC 5.4.2.8); 9: *mpg*-gene coding for a Mannose-1-phosphate guanyltransferase (EC 2.7.7.22)

The *gum*-like clusters of both *Kozakia* strains are separated from genes, which encode enzymes necessary for the synthesis of specific activated nucleotide precursors, including UDP-glucose, UDP-galactose and GDP-mannose. These genes are located at different genomic positions in both *K. baliensis* strains. These sequences were at first automatically annotated in the course of genome annotations and afterwards assigned to a proposed biosynthesis pathway for activated nucleotide sugars, which again was reconstructed from schemes involving essential enzymes for the respective biosynthesis of activated sugars [26, 18] (Fig. 4).

### Enzymes and clusters coding for putative HoPS biosynthesis

**Fig. 5:**
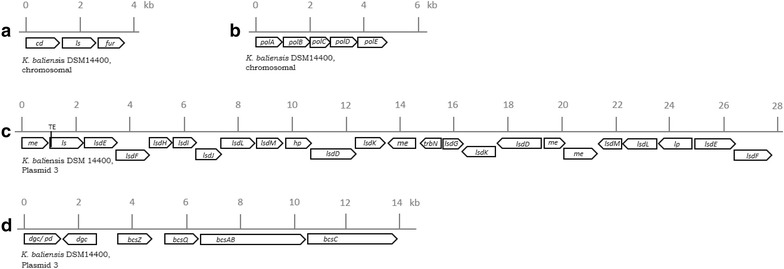
Overview of additional EPS forming enzymes and clusters in *K. baliensis*. Figure (**a**) shows the *levansucrase* genes (*ls*) (EC 2.4.1.10) of *K. baliensis* DSM 14400 and NBRC 16680, flanked by a cysteine desulfurase (EC 2.8.1.7) (*cd*) and a ferric uptake regulation protein (*fur*), respectively. In (**b**) the genetic organization of the chromosomally located *pol* cluster of *K. baliensis* DSM 14400 is exemplarily depicted. The pol cluster contains 5 genes (*polABCDE)*, which are functionally designated as *polA*: dTDP-glucose 4,6-dehydratase (EC 4.2.1.46), *polB*: glucose-1-phosphate thymidylyltransferase (EC 2.7.7.24), *polC*: dTDP-4-dehydrorhamnose 3,5-epimerase (EC 5.1.3.13), *polD*: dTDP-4-dehydrorhamnose reductase (EC 1.1.1.133), *polE*: alpha-L-Rha alpha-1,3-l-rhamnosyltransferase (EC 2.4.1.-). **c** Shows an additional plasmid located levansucrase gene (EC 2.4.1.10) of *K. baliensis* DSM 14400. It shares highest similarity to *Ga. diazotrophicus* levansucrase *lsdA* and is flanked by a (partial) type II dependent secretion operon [45]). In (**d**) is the genetic organization of the *K. baliensis* DSM 14400 cellulose synthase operon illustrated. The operon consists of six genes: *dgc/pd* diguanylate cyclase/phosphodiesterase (GGDEF & EAL domains) with PAS/PAC sensor(s), *dgc* diguanylate cyclase, *bcsZ* endoglucanase precursor (EC 3.2.1.4), *bcsQ* NTPase, *bcsAB* cellulose synthase catalytic subunit [UDP-forming] (EC 2.4.1.12), *bcsC* cellulose synthase operon protein C

In both strains complete ORFs coding for levansucrases of the glycoside hydrolase 68 family (GH 68) could be detected. Both *K. baliensis* strains possess one chromosomally encoded levansucrase gene, respectively, which share identical sequences and exhibit highest similarities to *Ko. xylinus* levansucrase (AB034152) (Fig. 5a). *K. baliensis* DSM 14400 additionally harbors a plasmid-encoded levansucrase, which shares highest similarity to *Ga. diazotrophicus* levansucrase *lsdA* gene, but seems to be inactive (interrupted) due to the insertion of a mobile element in the N-terminal domain. Similarly to *Ga. diazotrophicus*, a (partial) type II dependent secretion operon is associated with the interrupted levansucrase in *K. baliensis* DSM 14400. Though, no levanase gene (*lsdB*) could be detected downstream of the (interrupted) *lsdA* gene of *K. baliensis* (Fig. 5c).

Moreover, we identified a cellulose synthase operon on plasmid 3 of *K. baliensis* DSM 14400 (pKB14400_3), including genes encoding the three cellulose synthase subunits A, B and C, as well as a diguanylate cyclase (DGC) and phosphodiesterase (Fig. 5d).

### Genetic characterization of a HePS deficient mutant of *K. baliensis* NBRC 16680

**Fig. 6:**
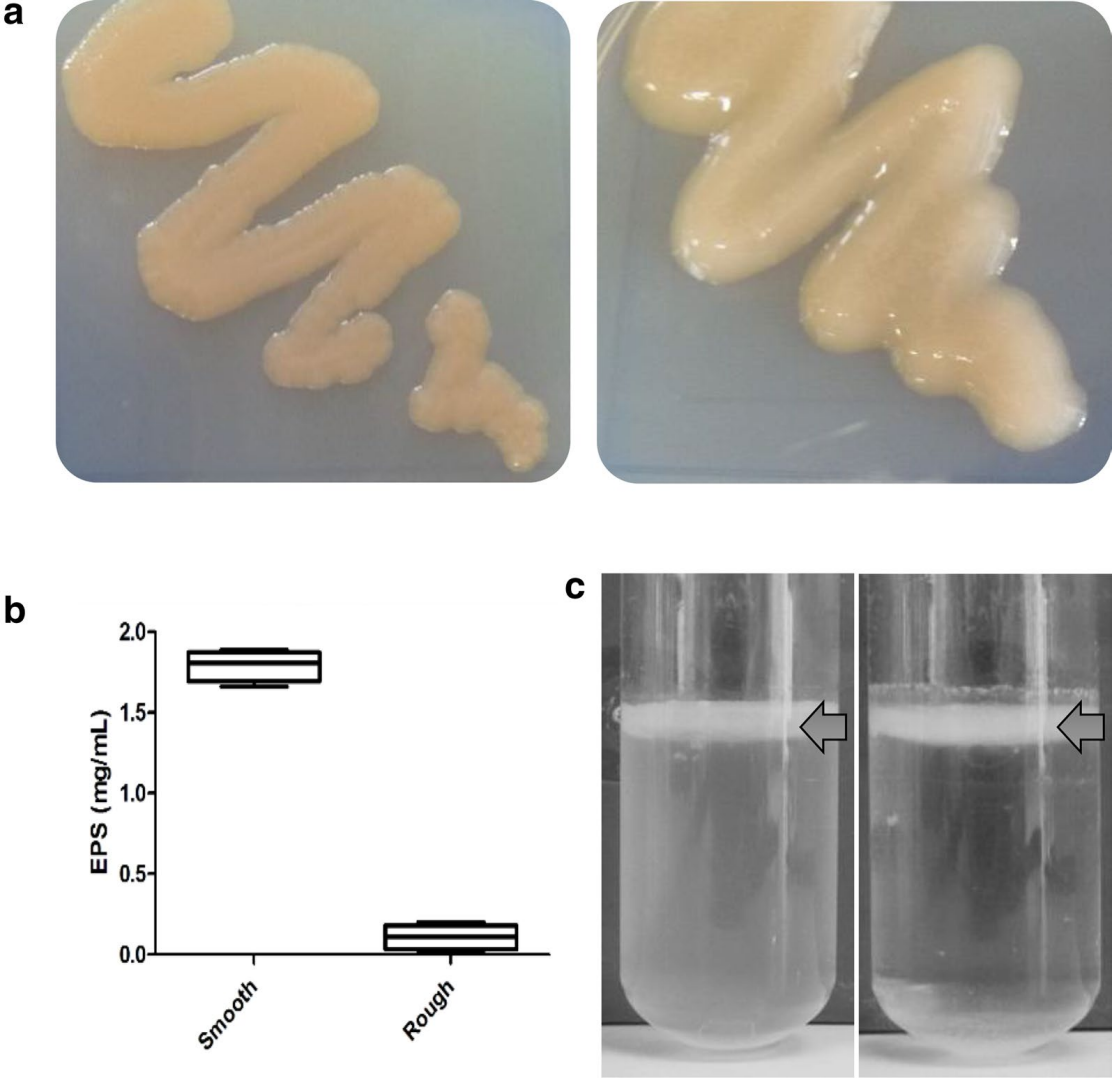
Comparison of *K. baliensis* NBRC 16680 wildtype and a HePS deficient mutant strain. **a** Phenotyps of *K. baliensis* NBRC 16680 rough (*left*, mutant) and smooth strain (*right*, wildtype); **b** isolatable polymeric substances from supernatants of *K. baliensis* NBRC 16680 wildtype strain (*left*) and *K. baliensis* NBRC 16680 mutant strain (*right*); **c** growth behavior of *K. baliensis* NBRC 16680 wildtype (*left*) and mutant strain (*right*) in static culture. The mutant strain exhibits a transposon insertion in its *gum*-like cluster (Fig. 7)

**Fig. 7:**
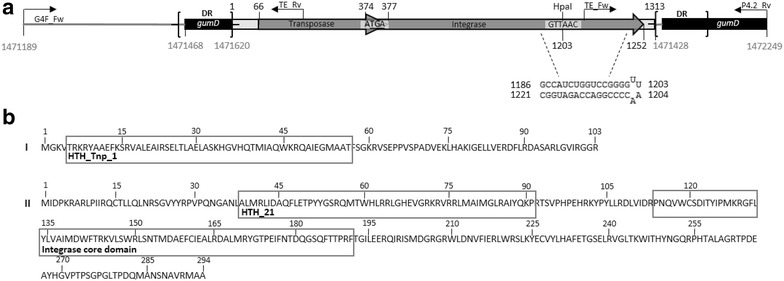
Schematic representation of the transposon insertion at the *gumD* locus in the HePS biosynthesis deficient mutant strain of *K. baliensis* NBRC 16680. **a** Transposon insertion locus at the *gumD* gene. The first 279 bp represent the upstream region of the *gumD* gene (1,471,189–1,471,468 bp). The transposon insertion caused a duplication in the insertion region [direct repeat sequence (DR)] comprising 40 bp of the upstream chromosomal region of the *gumD* gene, as well as a part of the *gumD* sequence itself (1,471,428–1,471,620 bp) (the DRs are highlighted by* black brackets*, respectively). The mobile element has a total length of 1313 bp, which includes an ORF coding for a putative transposase (66–377), an ORF coding for a putative integrase (374–1252) and flanking regions (1–66; 1253–1313). The nucleotide sequence of the mobile element (1313 bp) is identically present at three further genomic regions of the *K. baliensis* NBRC 16680 chromosome (77,364–78,676 bp, 1,430,134–1,428,821 bp, 2,279,289–2,280,601 bp). At positions 1186–1221 bp of the mobile element a putative stem loop structure (dyad symmetry: 29.8 kcal/mol) could be predicted, which contains the restriction site for HpaI (5′-GTTAAC-3′; further explanation in text). The depicted genomic area was amplified with primers G4F_Fw and P4.2_Rev.** b** In I the protein sequence of a putative transposase with a predicted HTH_Tnp1 helix turn helix (bit score: 41.95, E-value: 5.57e-07) is depicted; II shows the protein sequence of a putative integrase and its predicted functional domains: HTH-like helix-turn-helix domain (bit score: 57.18, E-value: 1.44e-11) and integrase core domain (bit score: 104.65, E-value: 1.42e-28)

We identified a spontaneous mutant of *K. baliensis* NBRC 16680, which exhibited an altered rough colony morphology on solid NaG agar and was not able to secrete HePS in shaking cultures in contrast to the wildtype strain (Fig. 6a, b). Nevertheless, both types were still able to form a pellicle, floating on the media surface of a static culture (Fig. 6c), which was shown to be dependent of functional Pol proteins in *A. tropicalis* [24]. To identify possible mutations in the respective *pol* (Fig. 5b) and *gum*-like clusters of *K. baliensis* NBRC 16680, PCR screenings covering these genomic regions were performed, respectively. No mutations were observed in the *pol*-clusters of the mutant strain. The 25 kb *gum*-like cluster was divided into six segments (approximate sizes of about 4–5 kb), which were amplified via PCR reactions (primers listed in Additional file 1: Table S1). In segment four, which includes two hypothetical proteins (1,466,953–1,467,825 bp, 1,469,289–1,467,916), an oxidoreductase (1,469,488–1,470,672 bp) and a part of the *gumD* gene (1,471,468–1,472,248 bp), a larger PCR product as expected (6100 bp) was observed in case of the mutant strain. Sanger sequencing of the larger PCR amplicon yielded no positive results, possibly due to a transposon insertion, leading to a hairpin loop formation, which could hinder sequencing [32]. Therefore, several restriction enzymes were tested to perform a single cut in the larger PCR product of the rough strain for possible interruption of the loop structure of a putative transposon. Restriction of the larger PCR product with HpaI allowed sequencing of the obtained restriction fragments (possibly because of disruption of an energy-rich stem loop structure) with primers G4F_Fw and P4.2_Rv (Fig. 7). In this way, a transposon insertion in front of the *gumD* gene of the rough strain could be identified, as well as the transposon itself. Via a further PCR reaction with specific transposon specific primers in both directions (TE_Fw & TE_Rv), the exact location of the transposon insertion could be identified. The transposon is located in front of the *gumD* gene, while a short region of the starting sequence of *gumD* had duplicated (direct repeat, DR). The transposon insertion possibly leads to an interruption of the promotor region of these gene and, subsequently, a total inactivation of HePS production and secretion (Fig. 6b). The identical nucleotide sequence of the mobile element could be detected thrice in the genome of *K. baliensis* NBRC 16680, each chromosomally located: 77,364–78,676 bp, 1,430,134–1,428,821 bp, 2,279,289—2,280,601 bp. The transposon shows furthermore high similarity to parts of a putative transposon of *G. oxydans* 621H (GOX1325) [Identities: 771/827(93 %), Query: (98 %)].

## Discussion

Acetic acid bacteria are well known to produce relatively large quantities of EPS. These can be either HoPS or HePS, but only a few AAB are noted to produce both kinds of the EPS simultaneously. Strains of *Ko. xylinus* (formerly *Ga. xylinus*) produce for example a water insoluble cellulose, levan as well as a water soluble HePS named acetan [17, 10, 18]. *K. baliensis* is a new candidate within the family of AAB that was investigated in this study to produce as well multiple EPSs.

### Characterization of novel HePS from *K. baliensis* via comparative genome and sugar monomer analyses

*Kozakia (K.) baliensis* DSM 14400 is known to produce high amounts of high molecular weight levan with sucrose as the main carbon source [19, 20]. Besides the well-studied levan, we elucidated the identity and the genetic background of so far not characterized HePS from two *K. baliensis* strains in this work, via sugar monomer analysis of isolated EPS and genome sequencing/EPS biosynthesis cluster annotation of/in the respective strains. Both EPS consist of glucose, galactose and mannose. Via comparison of the genomes of *K. baliensis* with related AAB and comprehension of known literature about EPS biosynthesis in AAB and the commercial xanthan producer *X. campestris*, we were able to get new insights into HePS biosynthesis in *K. baliensis*. The identified *gum*-like clusters from *K. baliensis* are highly similar to other HePS producing AAB, like *Ga. diazotrophicus* PA15 (Fig. 3b) and *Ko. xylinus* E25 (Fig. 3c). *Ga. diazotrophicus* harbors – in comparison to *Kozakia*—the most related HePS biosynthesis cluster, which also includes several *gum*-like genes (*gumB, gumC, gumD, gumE, gumH, gumJ, gumK* & *gumM*, derived from the well characterized xanthan biosynthesis) and produces a HePS that consists of d-glucose, d-galactose and d-mannose as well [31]. *Ko. xylinus* produces “acetan”, which consists of d-glucose, d-rhamnose, d-mannose and d-glucuronic acid [10, 33, 34]. Via comparison between both *K. baliensis* clusters and the above mentioned AAB clusters with the well characterized *gum*-cluster of *X. campestris*, genes could be identified, which code for enzymes that are at least necessary for incorporation of some of the HPLC analyzed monomers glucose and mannose into the HePS of *K. baliensis* DSM 14400/NBRC 16680 (Table 2). Though it has to considered, that some further present sugar transferases with unknown function could catalyze the incorporation of these sugar monomers at certain positions with certain linkage types. Since the HePS from *X. campestris* and *Ko. xylinus* do not contain galactose [17], one or more of the homologous glycosyltransferases solely present in *K. baliensis* and *Ga. diazotrophicus gum*-like clusters could function as specific galactosyltransferases (Fig. 3). Additionally, further differences among the compared AAB *gum*-like clusters could be identified (Fig. 3). The *Ko. xylinu*s E25 “acetan” cluster is flanked by a mannose-1-phosphate guanylyltransferase *manB*, which is separately located from the HePS cluster in both *K. baliensis* genomes. In contrast to *K. baliensis,* a *gumJ* gene, necessary for the translocation of the HePS across the membrane [11], is missing in the acetan cluster. However, this could be functionally replaced by the exclusively in the acetan cluster present *aceC* gene, which contains a MATE Wzx-like domain, that could act as a flippase (see also ''Background'' section), assisting the membrane translocation of acetan. Via homology comparison between the *aceP* and *aceQ* gene of the *Ko. xylinu*s E25 cluster with the glycosyltransferase 1 and 2 genes of both *K. baliensis* strains (Fig. 3), these could be identified as additional glucosyltransferases, as shown for *Ko. xylinus* by Ishida T, Sugano Y and Shoda M [35]. Furthermore, the cluster from *Ko. xylinus* involves a rhamnosyltransferase gene (*aceR*) as well as a *gumF* like gene (Fig. 3), coding for a protein that incorporates acetyl residues, which both are not included in the respective *K. bali*ensis clusters. This is in agreement with the structural analysis of both *K. balie*nsis HePS, which contain no rhamnose, while the missing of acetyl-groups in both *Kozakia* HePS has still to be investigated. On the contrary, the most similar *gum*-like cluster of *Ga. diazotrophicus* (in comparison to *Kozakia*) also includes the putative acetyltransferase *gumF*, indicating additional acetyl residues. This shows that - although the *gum*-like HePS clusters of *K. baliensis* and *Ga. diazotrophicus* are similarly organized, most of the respective encoded proteins exhibit relatively high homology among each other and HPLC analysis revealed these EPS to be composed of the same sugar monomers—the differences in specific gene sets could lead to specific structures and properties of these EPS. These observations nicely demonstrate the high value of the sequencing based function prediction regarding the investigated gum-like clusters. The detection of a *gumK* gene in both *K. baliensis* clusters for example suggests the existence of an additional glucuronic acid (GlcA) residue, which could not be detected via HPLC sugar monomer analysis, while preliminary NMR analyses confirmed this prediction (data in progress). This shows that genomic analysis, as an interposed first step, can successfully support the prediction of complex HePS structures.

The active expression of the HePS clusters from *K. baliensis* was most likely proven via a mutant (R-strain) in an essential HePS-formation associated gene (*gumD*, Fig. 6). This mutant strain of *K. baliensis* NBRC 16680 is not the result of an aberration in *polABCDE* cluster (Fig. 5b) as demonstrated for *A. tropicalis* [24]. Amplification and sequencing of the *pol* clusters of the R strain showed no aberration in comparison to the S (wildtype) strain. Since both the R- and the S-strain are still able to form a surface pellicle, the synthesis of secreted HePS by *K. baliensis* is unlinked with pellicle formation [24]. Via PCR screening of the gum-like cluster of *K. baliensis* a transposon insertion in front of the *gumD* gene was found (Fig. 7). This gene catalyzes the first step of HePS synthesis in *X. campestris*, by transferring the a glucosyl-1-phosphate residue from UDP-glucose to an undecaprenyl phosphate residue. A mutation in this gene leads to a lack in xanthan synthesis by *X. campestris*, confirming the essential function of this protein during xanthan synthesis [28]. Therefore, a similar function can be assumed in the case of *K. baliensis* NBRC 16680.

### Multiple EPS production by *K. baliensis* and its possible role in the environment

In addition to the *gum*-like and *pol* clusters, we were able to identify chromosomally encoded levansucrases in both investigated *Kozakia* strains. Further HoPS forming enzymes were detected on plasmid 3 of *K. baliensis* DSM 14400 [a cellulose synthase operon and a levansucrase flanked by a type II secretion operon with high similarity to a *Ga. diazotrophicus* levansucrase (GDI_RS02220)], both of which are framed by mobile elements. In contrast to the cellulose synthase operon, including all necessary elements for cellulose formation [36], the levansucrase on plasmid 3 is interrupted by a transposon insertion and accordingly inactive. This collection of genes connected to polysaccharide formation therefore appears to provide an evolutionary and ecological advantage and possibly leads to a high physiological adaption of this strain to its environment. The additional production of other/further EPS like cellulose may lead to further advantages in changing environments. The production of cellulose is mostly known from *Komagataeibacter* and *Acetobact*er, which prefer alcohol-enriched environments. These bacteria grow in cellulose surface pellicles that-apart from floating on surfaces for sufficient oxygen supply due to their strictly aerobic metabolism—most likely function as a barrier and protect them against osmotic stress caused by alcohol [37]. It seems that by randomly up taking EPS sequences from related AAB, *K. baliensis* collects EPS forming genes that are not necessarily active, like the levansucrase on plasmid 3 of the DSM 14400 strain, but may be activated in times of environmental changes. Related bacteria (especially regarding EPS synthesis as demonstrated in this study) such as *Ga. diazotrophicus* use these EPS for the protection against abiotic or biotic factors like desiccation and osmotic stress [38]. *Ga. diazotrophicus* is a nitrogen-fixing, endophytic bacterium, known to symbiotically colonize plants like sugar-cane [39], rice [40], as well pineapple [41] via production of levan from sucrose [42]. Because of the fact, that both *K. baliensis* genomes do not contain any nitrogen fixing genes in contrast to *Ga. diazotrophicus*, it is possible that instead of playing a role as an essential nitrogen fixing endosymbiont, *K. baliensis* uses EPS such as HePS or levan to colonize plants without positive influence on the respective plants. The association of *K. baliensis* with plant material is furthermore indicated by endoglucanases in the gum-like clusters (Fig. 3a), which could play a role during plant cell wall degradation [43]. Some other AAB strains are well known to cause fruit infections like the so-called pink disease in pineapples that results in pinkish discolorations [4]. Moreover, *X. campestris* uses xanthan as a virulence factor during infection of diverse plants. Chou et al. demonstrated that disruption of *gumD* leads to a reduced virulence of *X. campestris* in case of black rot in *Brassica oleracea*. This implicates the importance of specific EPS during microbe-plant interactions, while any involvement of EPS from *K. baliensis* in plant pathogenicity/colonization still remains to be demonstrated.

## Conclusions

Via a comparative genomic approach we could get new valuable insights into the biosynthesis of novel HePS produced by *K. baliensis*, which is related to the biosynthesis of the biotechnologically widely used HePS xanthan. Though, the properties of these novel HePS might remarkably differ from xanthan due to their different sugar/acid compositions pointing out the uniqueness of these novel HePS. The genomic approach applied in this work is enormously time saving and efficiently supports future chemical analyses such as NMR for final elucidation of these complex HePS structures. The obtained data can be used for the knowledge-based optimization and engineering of HePS production by *K. baliensis* via specific characterization of enzymes involved (identification of specific enzyme functions via activity assays and generation of deletion mutants) and transcriptional/proteomic studies.

## Additional files

10.1186/s12934-016-0572-x Primers used for transposon identification in *K. baliensis* NBRC16680.

10.1186/s12934-016-0572-x Enzymes related to the EPS nucleotide sugar biosynthesis in *K. baliensis* (DSM 14400, NBRC 16680) and their genetic location.

## Abbreviations

AAB: acetic acid bacteria
BLASTP: basic local alignment search tool
DGC: diguanylate cyclase
DSM: Deutsche Sammlung von Mikroorganismen
EPS: exopolysaccharide
Gal: galactose
GH 68: glycoside hydrolase 68 family
Glc: glucose
GT: glycosyltransferases
gum: protein involved in HePS biosynthesis (derived from *X. campestris*)
HePS: heteropolysaccharide
HGAP: hierarchical genome-assembly process
HoPS: homopolysaccharide
HPLC: high-performance liquid chromatography
K.: *Kozakia*
Ko.: *Komagataeibacter*
Man: mannose
MWCO: molecular weight cut-off
NaG: modified sodium-gluconate medium
NBRC: Biological Research Centre NITE, Japan
ORF: open reading frame
Pol: protein involved in pellicle formation
RI: refractive index
SMRT: single-molecule real-time sequencing
X.: *Xanthomonas*
