# Supplementary material for: Dissection of exopolysaccharide biosynthesis in Kozakia baliensis
Source: Microb Cell Fact. 2016 Oct 4;15:170. doi: 10.1186/s12934-016-0572-x (PMC5050591; doi:10.1186/s12934-016-0572-x)
Supplement: Supplementary file 1 [file 12934_2016_572_MOESM1_ESM.docx]

Primer	Sequence (5`-> 3`)	primer location NBRC16680 genome	Product size
P1.1_Fw	GACCGAGCGTCCAGTTATTC	(1.456.282 – 1.456.302 bp)	Segment 1<br>4001 bp
P1.2_Rv	GGATGCTGCGCACTAATGGC	(1.460.262 – 1.460.282 bp)	Segment 1
P2.1_Fw	CGCCATTAGTGCGCAGCATC	(1.460.262 – 1.460.282 bp)	Segment 2<br>4009 bp
P2.2_Rv	CGATGCTGGTGCGGGTAATG	(1.464.250 – 1.464.270 bp)	Segment 2
P3.1_Fw	CATTACCCGCACCAGCATCG	(1.464.551 – 1.464.571 bp)	Segment 3<br>3310 bp
P3.2_Rv	CGGTCTTCAATCCTGTTCTG	(1.467.540 – 1.467.560 bp)	Segment 3
P4.1_Fw	CAGGATTGAAGACCGTACTC	(1.467.246 – 1.467.266 bp)	Segment 4<br>4703 bp
P4.2_Rv	AAACGGGTCCTTTCGAGTCC	(1.472.228 – 1.472.248 bp)	Segment 4
P5.1_Fw	TCTTCCGGCAACCGCGTATC	(1.472.249 – 1.472.269 bp)	Segment 5<br>4016 bp
P5.2_Rv	GATCGGCGTCGTTCCAACTG	(1.476.244 – 1.476.264 bp)	Segment 5
P6.1_Fw	TCGCGTGCAGTTGGAACGAC	(1.476.238 – 1.476.258 bp)	Segment 6<br>2617 bp
P6.2_Rv	ACATCACCAGCACGTAGAAC	(1.478.834 – 1.478.854 bp)	Segment 6
TE_Fw	ACCAGATGGCCAACAGCAAC	transposon insertion	
TE_Rv	GATTGAGCGATCATCGTCTG	transposon insertion	
TE_A_Fw	GGCCGTCCGACCATTCAATC	(77.284 – 77.303 bp)	With TE_Rv<br>198 bp
TE_B_Fw	AAACCGCCTTGGCCGTTCTG	(1.430.163 – 1.430.144 bp)	With TE_Rv<br>147 bp
TE_C_Fw	ACGCCACATAATCCGCATAG	(2.279.157 – 2.279.176 bp)	With TE_Rv<br>250 bp
G4F_Fw	ACGACGCGCATGAGTAAACC	(1.471.189 – 1.471.208 bp)	

Supplementary file 1. Primers used for transposon identification in *K. baliensis* NBRC16680
