# Supplementary material for: Dissection of exopolysaccharide biosynthesis in Kozakia baliensis
Source: Microb Cell Fact. 2016 Oct 4;15:170. doi: 10.1186/s12934-016-0572-x (PMC5050591; doi:10.1186/s12934-016-0572-x)
Supplement: Supplementary file 2 [file 12934_2016_572_MOESM2_ESM.docx]

Supplementary file 2. Enzymes related to the EPS nucleotide sugar biosynthesis in  K. baliensis (DSM 14400, NBRC 16680) and their genetic location .
